# Post-Traumatic Osteoarthritis in Mice Following Mechanical Injury to the Synovial Joint

**DOI:** 10.1038/srep45223

**Published:** 2017-03-27

**Authors:** Muhammad Farooq Rai, Xin Duan, James D. Quirk, Nilsson Holguin, Eric J. Schmidt, Nobuaki Chinzei, Matthew J. Silva, Linda J. Sandell

**Affiliations:** 1 Department of Orthopaedic Surgery, Musculoskeletal Research Center, Washington University School of Medicine at Barnes-Jewish Hospital, St. Louis, MO, USA; 2 Department of Cell Biology and Physiology, Washington University School of Medicine at Barnes-Jewish Hospital, St. Louis, MO, USA; 3 Mallinckrodt Institute of Radiology, Washington University School of Medicine, St. Louis, MO, USA; 4 Department of Biomedical Engineering, Washington University in St. Louis, St. Louis, MO, USA; 5 Department of Physician Assistant Medicine, School of Graduate Health Studies, Lynchburg College, Lynchburg, VA, USA

## Abstract

We investigated the spectrum of lesions characteristic of post-traumatic osteoarthritis (PTOA) across the knee joint in response to mechanical injury. We hypothesized that alteration in knee joint stability in mice reproduces molecular and structural features of PTOA that would suggest potential therapeutic targets in humans. The right knees of eight-week old male mice from two recombinant inbred lines (LGXSM-6 and LGXSM-33) were subjected to axial tibial compression. Three separate loading magnitudes were applied: 6N, 9N, and 12N. Left knees served as non-loaded controls. Mice were sacrificed at 5, 9, 14, 28, and 56 days post-loading and whole knee joint changes were assessed by histology, immunostaining, micro-CT, and magnetic resonance imaging. We observed that tibial compression disrupted joint stability by rupturing the anterior cruciate ligament (except for 6N) and instigated a cascade of temporal and topographical features of PTOA. These features included cartilage extracellular matrix loss without proteoglycan replacement, chondrocyte apoptosis at day 5, synovitis present at day 14, osteophytes, ectopic calcification, and meniscus pathology. These findings provide a plausible model and a whole-joint approach for how joint injury in humans leads to PTOA. Chondrocyte apoptosis, synovitis, and ectopic calcification appear to be targets for potential therapeutic intervention.

Osteoarthritis (OA) is a clinical syndrome of joint pain accompanied by varying degrees of functional impairment and diminished quality of life. OA represents the most common form of arthritis and it is estimated that by 2030, 67 million Americans (nearly one-third of adults ages 45–64 years) will have arthritis^1^. OA has raised the annual aggregated medical expenditures by $185.5 billion^2^. OA is a progressive disease and its progression eventually leads to significant disability often requiring costly arthroplasty, as there are currently limited medical treatments and no disease-modifying OA drugs. OA is defined as cartilage loss and osteophyte formation. Primary age-related OA has an unknown etiology due to a complex and multifaceted pathogenesis and an uncertainty about the timeline of pathological events^3^. In contrast, post-traumatic OA (PTOA) that constitutes at least 12% of knee OA has an injury component associated with it where the disease-initiating injury event is known and sequelae of pathological footprints can be traced. Any injury severe enough to cause anterior cruciate ligament (ACL) tear and/or meniscus destabilization frequently leads to PTOA^4^,^5^,^6^. A consensus has been developed that if we wish to replicate an injury setting in rodents similar to humans, we must consider using a non-invasive injury model that avoids surgery in order to more closely mimic typical knee trauma in humans^7^,^8^,^9^,^10^,^11^. A non-invasive injury model of tibial compression−such as the one developed by us^10^ and others in a modified form^7^ −is attractive for studying PTOA in mice. Although, commonly referred to as “tibial compression” because of its use in understanding the adaptation of the tibia to mechanical forces in fact the whole knee is being subjected to an external force. This model is simple to implement, highly reproducible and offers tailoring of injury severity by varying loading intensity and duration. While each non-invasive model is a representative of specific conditions leading to OA, our model accurately recapitulates the mechanically induced mechanisms involved in injuries leading to PTOA in humans. This is mainly because joint pathology is initiated through direct damage to articular cartilage, bone, or soft tissue structures (ligaments and meniscus) of the joint as well as through destabilization of the joint after ACL tear.

ACL tears are one of the most frequent forms of knee ligament injury with an estimated 150,000–200,000 ACL tears occurring annually in the United States alone^12^. ACL tears have critical implications for the physiological biomechanics of the tibiofemoral joint, often leading to functional instability, damage to meniscus and articular cartilage and an increased risk for PTOA^5^,^13^,^14^. Consequently, a substantial proportion of ACL tears undergo surgical treatment. Since the ACL has limited healing capability^15^,^16^,^17^, the surgical procedure of choice is ACL reconstruction with upwards of 175,000 ACL reconstruction surgeries performed annually in the United States^18^. The projected timeline between occurrence of ACL tear and development of PTOA in humans is 10–20 years^19^ indicating that changes in the knee joint take place at the time of injury, which ultimately lead to radiographic OA. With this in mind, we undertook the current study to evaluate the progression of PTOA-like features in mice undergoing different mechanical loading regimens at early (up to day 14) and late (up to day 56) time points.

In this study, we aim to investigate the spectrum of lesions typical of PTOA in the articular cartilage, meniscus, synovium, ligaments, and bone in response to mechanical knee injury in an attempt to understand early and late events in PTOA. We hypothesize that alteration in murine knee joint following mechanical compression reproduces the molecular and structural features of human PTOA in degrees depending on load intensity and time.

## Methods

### Mice

Eighty-eight male mice from LGXSM-6 (n = 44) and LGXSM-33 (n = 44) strains were used. Mice were raised under appropriate standards of animal husbandry. Washington University Institutional Animal Care and Use Committee approved this study (Approval #20150224). All experiments were performed in accordance with relevant guidelines and regulations. Since we did not observe any significant differences between LGXSM-6 and LGXSM-33, therefore, we combined the data from these two strains to examine the response of the knee joint to mechanical injury.

### Knee injury induction

The right knee underwent one of three separate loading forces following our established procedure^10^,^20^,^21^. Briefly, mice were anesthetized using 2.5% isoflurane inhalation in 4-L/min of oxygen. The right tibia was positioned vertically, with the ankle upward and the knee downward in deep flexion between loading cups. Using a materials testing machine (InstronElectroPulse E1000), axial compression was applied through the knee via the upper cup, while the lower cup was fixed and linked to the load cell. Sixty cycles of the following pattern were applied: loading (either 6N, 9N or 12N) for 0.34 sec, rise and fall time of 0.17 sec and a baseline hold time of 10 sec. The ACL tear was confirmed by the release of compressive force during compression. Mice were allowed to progress for 5, 9, 14, 28, or 56 days after loading (n = 8 each loading and time point). Contralateral left limb served as a non-loaded control. We selected early time points for 6N loading (5, 9, 14 days) as we did not expect severe consequences with 6N loading based on preliminary studies, late time points for 12N loading (12, 28, 56 days) and early and late time points for 9N loading (5, 9, 14, 28, 56 days). Mice were sacrificed using carbon-dioxide asphyxiation at the indicated time points. The knee joints were harvested and extra-articular soft tissues were removed, taking care not to violate the joint capsule. Knee joints were then fixed in 10% neutral buffered formalin (Sigma-Aldrich).

### Micro-CT

Prior to decalcification, knees were scanned using vivaCT-40 micro-CT scanner (Scanco-Medical) for analysis of 3-dimensional bone structure for various parameters at given settings^22^,^23^: voxel size = 21 μm, energy = 45  kV, intensity = 177 μA and integration time = 300  ms. The following morphometric parameters of the tibial cancellous bone were calculated for trabecular compartments: trabecular bone volume fraction (BV/TV), trabecular thickness (Tb.Th), trabecular separation (Tb.Sp), trabecular number (Tb.N), connectivity density index (CDI), structure model index (SMI) and apparent bone mineral density (BMD). Subchondral bone thickness was measured using a custom-written MATLAB-2015b (Mathworks) program^24^. Ectopic calcified nodules were visualized by constructing 3-dimensional images in OsiriX imaging software v.7 (OsiriX). Calcified nodules were quantified by including all mineralized areas in and around the joint space excluding the patella, anterior horns of the menisci and fabella^7^. All analyses were performed in a blinded fashion.

### MRI

*Ex vivo* MRI was performed prior to decalcification. Following fixation, knees were equilibrated in 5 mM Gd-DTPA (Magnevist, Berlex Imaging) for a minimum of 48 h and embedded in 2% agar gel. Both knees were simultaneously imaged on an Agilent 11.7 T Direct Drive MRI system using a 1 cm surface coil. T1-weighted 3-dimensional gradient echo images were acquired at 50 μm isotropic resolution within 2 h using these parameters: TR/TE = 25/2.2 ms, flip angle = 50 degrees, 6 averages. Each knee was independently scored for damage to the ACL, PCL (posterior cruciate ligament), and meniscus. For ACL and PCL pathology, a score of zero points was assigned when the ligaments appeared as a continuous band of uniform signal intensity, a score of 1 points was ascribed when the band appeared thin but continuous and a score of 2 points was given when the band appeared thin and broken with varying intensities. For meniscus pathology, a score of 0 points was given when it appeared normal, a score of 1 points was assigned when it appeared somewhat abnormal, and a score of two points was ascribed when meniscus appeared abnormal in shape concomitant with tear, extrusion, or edema. For ectopic calcification and osteophyte formation, the counts of calcified nodules and number of osteophytes were counted respectively. All scoring was carried out in a blinded fashion.

### Histology

Knees were decalcified in 5% formic acid (Decal Chemical Corp.) and processed for standard paraffin embedding. Five-micrometer thick serial sagittal sections were cut extending through the entire joint width. Every 10^th^ section was stained with Safranin-O to evaluate cartilage proteoglycan^7^,^9^. The cover-slipped sections were imaged using NanoZoomer digital slide scanner (Hamamatsu) and cartilage injury was identified. Using three selected Safranin-O stained sections synovitis was evaluated as before^25^.

### Apoptosis

*In situ* detection of chondrocyte apoptosis was undertaken using the terminal deoxynucleotidyltransferase-mediated dUTP nick-end labeling (TUNEL) assay (Roche). TUNEL-positive cells were counted in non-calcified cartilage^26^ since calcified cartilage naturally develops apoptosis^27^,^28^.

### Aggrecan expression

Antigen retrieval was achieved with 10 μg/mL proteinase K (EMD Millipore) in 10 mM Tris-HCl (pH 7.4–8.0) for 20 min at 37 °C. Auto-fluorescence was quenched with 50 mM NH_4_CI while non-specific binding was blocked with 10% goat serum for 1 h at room temperature. Slides were incubated with rabbit anti-aggrecan (G2 domain, 1:100) in 2% goat serum overnight at 4 °C in a humidified chamber. Antigens were detected by Alexa Fluor 594 conjugated goat anti-rabbit polyclonal antibody (Abcam, 1:200) for 1 h at room temperature. Sections were mounted with VECTASHIELD Mounting Medium with DAPI (VectorLab). Images were captured on an Eclipse E800 microscope (Nikon) with QImaging Retiga 2000R Fast 1394 camera and MetaMorph v7.7 software (Molecular Devices).

### Expression of type I collagen and type II collagen

For immunostaining of type I collagen and type II collagen, slides were incubated with rabbit polyclonal antibody to type I collagen (ab 34710; Abcam) or with antibody IIF (that recognizes the triple-helical domain of type II collagen protein)^29^ at 1:200 dilutions in 2% goat serum at 4 °C overnight. The secondary antibodies used were goat anti-rabbit Alexa Fluor 488 (ab150077, Abcam) and goat anti-rat Alexa Fluor 594 (ab150160, Abcam), respectively, at dilutions of 1:250 in 2% goat serum in PBS for 1 h at room temperature. Finally, the slides were mounted with VECTASHIELD Mounting Medium with DAPI (VectorLab). Images were captured as described above.

### Staining for ectopic calcification

Some knee joints were fixed in 10% neutral buffered formalin for 48 h and then kept in 70% ethyl alcohol until embedded in methyl methacrylate for plastic sections. To detect calcified nodules, 5 μm thick sections were incubated for 30 min with 5% aqueous silver nitrate solution in daylight using von Kossa method. Sections were then washed twice with distilled water, and then treated with 5% sodium thiosulfate. Counterstaining was performed with the use of 1% basic fuchsin solution. Images were taken as mentioned above.

### Statistical analysis

Data were analyzed using GraphPad Prism v6.04 (GraphPad Software Inc.) and are shown as mean ± standard deviation unless stated otherwise. A 2-way analysis of variance (ANOVA) with Tukey’s post-hoc was used. This model included loading force, time point, and non-loading control knees as variables. P < 0.05 was set for statistical significance.

## Results

### Ligament pathology

**Figure 1:**
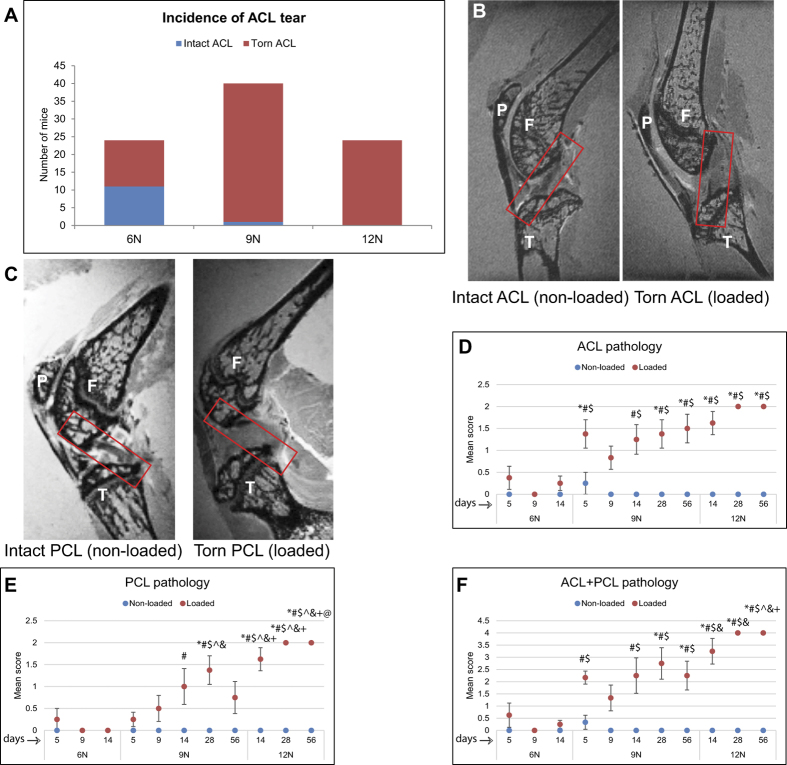
Ligaments pathology. The first evidence for the assessment of ACL tear was based on the release of compressive wave at the time of loading. We observed that the ACL tore more frequently at 9N (97.5%) and 12N (100%) than at 6N (54%) loading (**A**). ACL pathology was measured by MRI as well (**B**), left panel intact ACL, right panel torn ACL). The PCL pathology was also measured by MRI (**C**), left panel intact PCL, right panel damaged PCL). Quantification pathology of ACL (**D**), PCL (**E**) and ACL and PCL combined (**F**) showed that there was a significantly more damage to these ligaments between 6N and 9N groups at early time points and between 9N and 12N groups at late time points. ACL and PCL pathology was assessed on a three-tiered scale: 0 = ligaments appeared as a continuous band of uniform signal intensity, 1 = the band appeared thin but continuous and 2 = the band appeared thin and broken with varying intensities. F = femur, T = tibia, P = patella. *Compared to 6N 5 day, ^#^compared to 6N 9 day, ^$^compared to 6N 14 day, ^^^compared to 9N 5 day, & compared to 9N 9 day, @ compared to 9N, 14 day, ^+^compared to 9N 56 day.

We observed a load-dependent increase in incidence of ACL tear at the time of loading (Fig. 1A). While in 6N group only 54% of knees experienced an ACL tear, 9N and 12N groups had respectively 97.5% and 100% tear rate. MRI assessment showed that ACL (Fig. 1B) and PCL (Fig. 1C) in non-loaded knees were a continuous band of uniform signal intensity while in loaded knees they appeared as a thin and broken band with varying intensities. ACL (Fig. 1D) and PCL (Fig. 1E) pathology alone and combined (Fig. 1F) demonstrated a significant increase in 9N and 12N groups at day 14 compared to 6N group with no significant differences between 9N and 12N groups at this time point. In addition, there was a significant increase in ACL pathology in 9N group at day 5 compared to 6N group. PCL pathology and ACL + PCL pathology also significantly increased at day 56 between 9N and 12N groups. These results indicate that ligament pathology was effected at early time points (days 5 and 14) in 6N and 9N groups and at later point (day 56) in 9N and 12N groups.

### Articular cartilage changes

**Figure 2:**
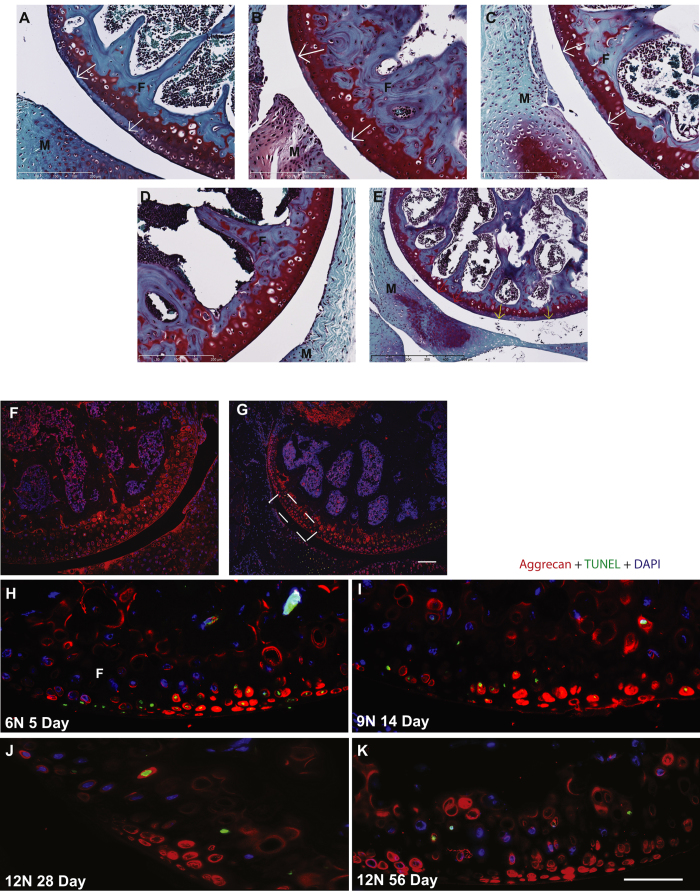
Cartilage injury, apoptosis, and distribution pattern of aggrecan protein. Loading introduced a primary cartilage injury on the posterior aspect of lateral femoral condyle identified by focal loss of Safranin-O staining (indicative of proteoglycan loss) and normal nuclear staining. We observed no difference in magnitude of primary injury with loading intensity or time point. Representative images of cartilage injury site are shown for 6N 5 days (**A**), bar = 200 μm), 9N 14 days (**B**), bar = 200 μm) and 12N 56 days (C, bar = 200 μm). Contralateral non-loaded knees were devoid of any cartilage injury (**D**), bar = 200 μm). The knees in which ACL was torn also showed a secondary injury distant from primary injury and was characterized by loss of proteoglycan (**E**, bar = 500 μm). We did not identify significant chondrocyte apoptosis using TUNEL assay (green staining) in the non-injured cartilage area (**F**) but observed significant apoptosis at the site of injury (**G**), white box, bar = 100 μm). We observed that apoptosis was present as early as day 5 after loading (**H**), decreased and diminished by day 14 (**I**) and absent at days 28 (**J**) and 56 (**K**) indicating that all cells in the impacted area undergo apoptosis and there is no progression over time or with loading (bar = 50 μm). Aggrecan (red staining) was found around the chondrocytes in the intact area and in the lacuna left by the apoptotic chondrocytes in the injured area (**G**–**I**). F = femur, T = tibia, M = meniscus.

The primary injury is mainly evident on the posterior aspect of lateral femoral condyle (Fig. 2A–C) due to a transient subluxation of the proximal tibia relative to distal femur resulting in ACL rupture as well as due to direct contact between tibia and femur from each loading cycle. Although an injury likely occurred on the tibia as well, we focused on the more reproducible, prominent and easily identifiable injury on the femur. Non-loaded control knees had no such injury (Fig. 2D). Secondary injury (Fig. 2E), at multiple adjacent sites, was located anterior to primary injury and was likely the outcome of continued compression following ACL/PCL rupture. Whether primary or secondary, each injury site was characterized by loss of Safranin-O staining (proteoglycan loss) and chondrocyte apoptosis. We observed no difference in cartilage damage or its progression to fibrillation and degeneration with time or loading, other than that explained by the ACL tear.

We observed no apoptotic chondrocytes in the non-loaded limbs (Fig. 2F, green). However, we observed chondrocyte apoptosis at the site of injury in loaded knees (Fig. 2G–K, green) as evidenced by TUNEL-positive (green) and DAPI-negative staining mainly at day 5. Numerous apparently empty lacunae, devoid of both TUNEL and DAPI staining, were found, which are likely remnants of the apoptotic chondrocytes. There was no difference in TUNEL-positive cells between loading intensities at day 5 (data not shown). There was no progression in or new development of apoptosis over the 56-day study period, therefore quantification of apoptosis was not possible at later time points. Instead, after day 9, virtually nuclei of all dead cells were cleared out and only a few, if any, apoptotic cells were randomly present and were mostly confined to the calcified cartilage. The pattern of aggrecan distribution was consistent across all time points and loading: increased presence of aggrecan protein (Fig. 2G–K, red) inside the lacunae at injury site and a smooth and intense pericellular distribution pattern in the non-injured area or in the non-loaded knees (Fig. 2F, red).

### Synovitis, meniscus pathology, and osteophytes

**Figure 3:**
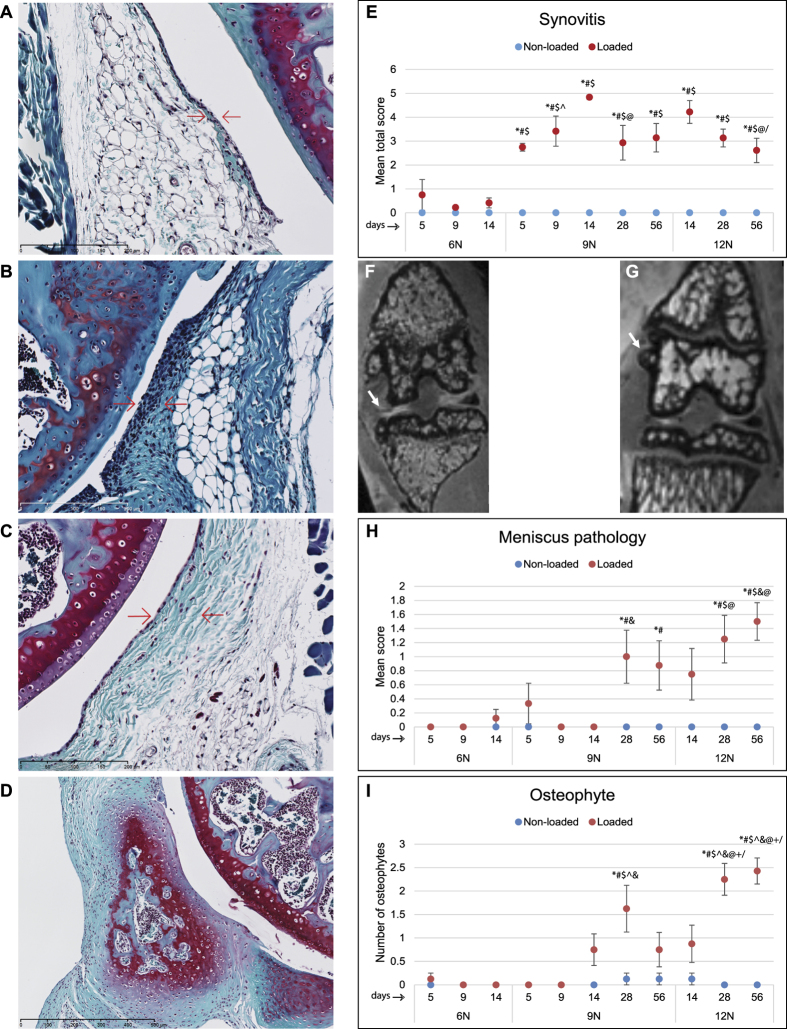
Synovitis, meniscus pathology, and osteophyte formation. Non-loaded knees showed normal synovial lining consisting of 1–2 cell layers (**A**), bar = 200 μm) while loading resulted in an enlargement of the synovial cell lining (**B**), bar = 200 μm). Similarly, in non-loaded knees there was normal density of the cells in the synovial stroma (**C**), bar = 200 μm) which increased with loading (**D**), bar = 500 μm). Quantification of synovitis for cell lining and cell density showed significantly higher synovitis in 9N group compared to 6N group at days 5, 9 and 14 (**E**). At day 28 in 9N group and at day 56 in 12N group, the synovitis was significantly less than at day 14 in 9N and 12N groups respectively. Synovitis remained low in 6N group, increased in 9N group up to day 14, and then decreased significantly by day 28. For 12N group, maximum synovitis was observed at day 14, which began to decrease by day 28, and significantly decreased at day 56 (**E**). MRI assessment of meniscus (arrow) (**F**) and osteophyte formation (arrow) (**G**) showed that meniscus pathology did not change significantly between 6N and 9N groups but it showed some changes between 9N and 12N groups at different time points (**H**). Similarly, quantification of osteophytes revealed that osteophyte count did not vary significantly between 6N and 9N groups, however there were significantly more osteophytes in 12N group than 9N group as well as these numbers increased significantly at day 56 compared to days 14 and 28 in 12N loading group (**I**). Meniscus pathology was assessed using this scoring system: 0 = normal, 1 = somewhat abnormal 2 = abnormal in shape concomitant with tear, extrusion, or edema. *Compared to 6N 5 day, ^#^compared to 6N 9 day, ^$^compared to 6N 14 day, ^^^compared to 9N 5 day, & compared to 9N 9 day, @ compared to 9N, 14 day, ^+^compared to 9N 56 day,/compared to 12N 14 day.

Synovium appeared normal in control knees (Fig. 3A,C). In loaded knees, there was substantially higher synovial cell proliferation and intimal lining cell hyperplasia (Fig. 3B,D). The degree of synovitis changed with both load intensity and time. For instance, at days 5, 9 and 14, there was a significantly higher synovitis in 9N group than 6N group (Fig. 3E). At day 28 in 9N group and at day 56 in 12N group, the synovitis was significantly less than at day 14 in 9N and 12N groups respectively. Synovitis remained low in 6N group, increased in 9N group up to day 14, and then decreased significantly by day 28. For 12N group, maximum synovitis was observed at day 14, which began to decrease by day 28, and significantly decreased at day 56. On MRI, meniscus pathology included abnormal meniscus shape, extrusion, displacement, tear, or edema (Fig. 3F). There were also osteophytes observed on MRI (Fig. 3G). The severity of meniscus pathology did not significantly changed between 6N and 9N groups but it did change between 9N and 12N groups at different time points (Fig. 3H). Similarly, osteophyte count did not vary significantly between 6N and 9N groups, however, there were significantly more osteophytes in 12N group than 9N group as well the numbers increased at day 28 compared to day 14 and at day 56 compared to both days 14 and 28 in 12N group (Fig. 3I) indicating a time- and load-dependent increase in the numbers of osteophytes.

### Trabecular bone parameters

**Figure 4:**
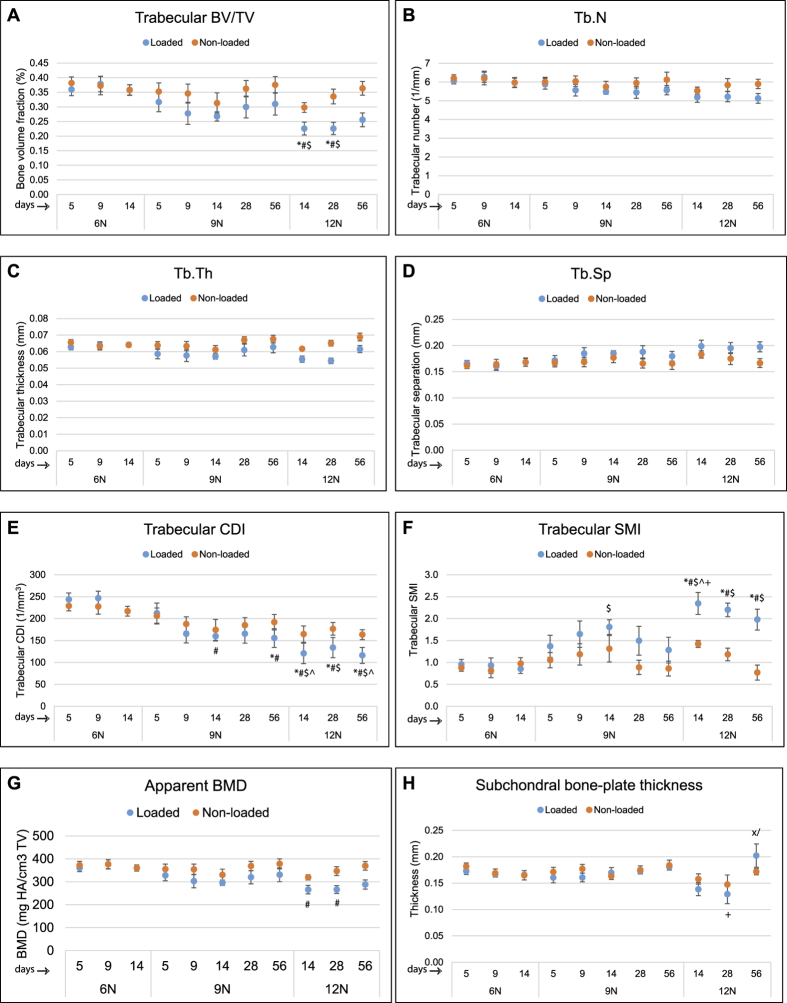
Trabecular bone measurements and subchondral bone plate thickness. Trabecular bone parameters measured in this study were derived from tibial epiphysis of non-loaded and loaded knees. Bone volume fraction (BV/TV) (**A**), trabecular number (**B**), trabecular thickness (**C**), trabecular spacing (**D**) and trabecular CDI (E) did not differ significantly between 6N and 9N groups at days 5, 9, and 14 or between 9N and 12N groups at days 14, 28 and 56. Trabecular SMI (**F**) increased significantly at day 14 in 9N and 12N groups than 6N group but did not change with time. There were no significant differences for apparent BMD (**G**). Coronal images were used for measurement of subchondral bone plate thickness in the medial and lateral tibial plateau. Subchondral bone thickness measurement showed that there were no significant differences in thickness of subchondral bone plate between non-loaded and loaded knees. However, there was a significant increase in subchondral bone plate thickness in 12N group at day 56 compared to days 14 and 28 (**H**). BV = bone volume, TV = tissue volume, Tb.N = trabecular number, Tb.Th = trabecular thickness, Tb.Sp. = trabecular spacing, CDI = connectivity density index, SMI = structure model index, BMD = bone mineral density. *Compared to 6N 5 day, ^#^compared to 6N 9 day, ^$^compared to 6N 14 day, ^^^compared to 9N 5 day, ^+^compared to 9N 56 day,/compared to 12N 14 day, x compared to 12N 28 day.

The changes in the different bone parameters are given in Fig. 4. Trabecular BV/TV (Fig. 4A), Tb.N (Fig. 4B), Tb.Th (Fig. 4C), Tb.Sp (Fig. 4D) and trabecular CDI (Fig. 4E) did not significantly differ between 6N and 9N groups (at days 5, 9 or 14) or between 9N and 12N groups (at days 14, 28 or 56). Trabecular SMI (Fig. 4F) was significantly increased at day 14 in 9N and 12N groups than 6N group but did not change with time. There was no significant difference between any two loading groups and time points for apparent BMD (Fig. 4G). Changes in subchondral bone plate thickness (Fig. 4H) became significantly apparent in 12N group when the thickness was significantly increased at day 56 compared to both days 14 and 28.

### Ectopic calcification

**Figure 5:**
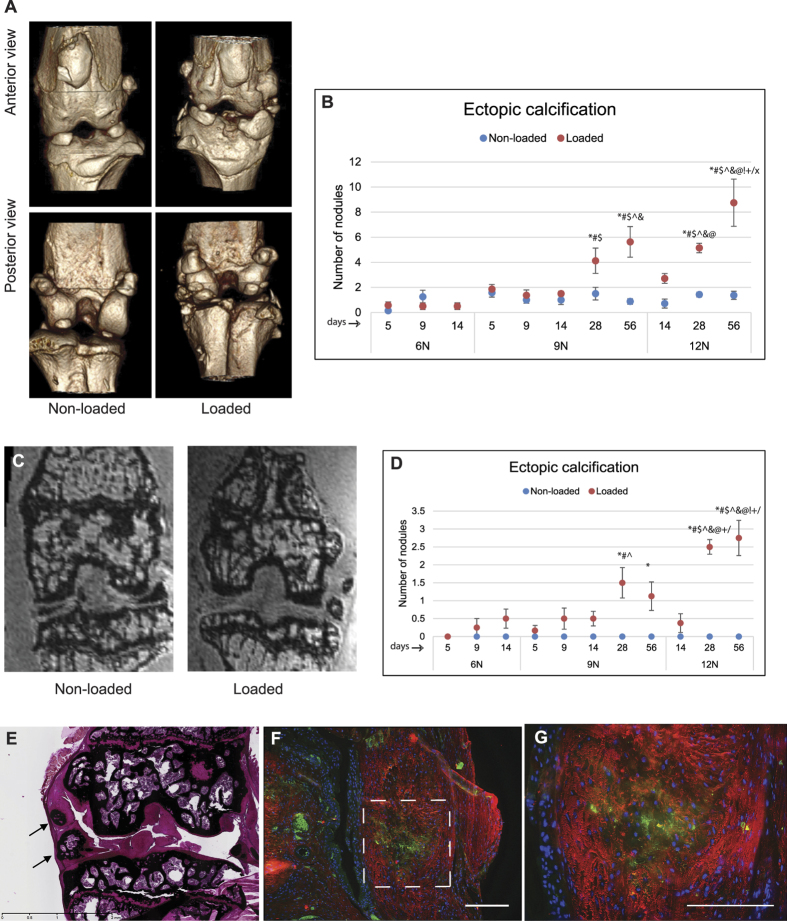
Ectopic calcification. Loaded knees developed periarticular calcified nodules (ectopic calcification). Representative micro-CT images (**A**) of ectopic calcification in non-loaded and loaded knees are shown. There was no significant difference in the number of calcified nodules (estimated by micro-CT) between 6N and 9N groups but there significantly more calcified nodules in 12N group compared to 9N group at day 56 and compared to 12N group at days 14 and 28 (**B**). Representative MRI images of ectopic calcification (**C**) along with counts of calcified nodules showed similar trend as that of determined by micro-CT assessment (**D**). The calcified nodules were positive for von Kossa staining (**E**). Immunostaining with type I (red) collagen and type II collagen (green) showed that center of the nodules was positive for type II collagen while periphery was positive for type I collagen as shown at lower (**F**) and higher (**G**) magnifications. Bar = 100 μm. *Compared to 6N 5 day, ^#^compared to 6N 9 day, ^$^compared to 6N 14 day, ^^^compared to 9N 5 day, & compared to 9N 9 day, @ compared to 9N, 14 day, ^!^compared to 9N 28 day, ^+^compared to 9N 56 day,/compared to 12N 14 day, x compared to 12N 28 day.

Micro-CT analysis revealed calcified nodules around joint capsule, synovium, or meniscus only in the knees that underwent loading (Fig. 5A). Unlike osteophyte that is attached to the bone surface, ectopic calcified nodules were embedded in the synovium and/or joint capsule. The nodules were rarely present in the non-loaded knees. The development of (synovial) ectopic calcification as determined by micro-CT showed no significant changes between 6N and 9N groups (Fig. 5B). The presence of nodules was also confirmed by MRI (Fig. 5C), which also showed no significant difference between 6N and 12N groups (Fig. 5D). However, significant increase in severity of ectopic calcification with time and loading was apparent in 9N and 12N groups (Fig. 5D). There was significantly higher number of calcified nodules (as measured by micro-CT) in 12N group at day 56 compared to 9N group at day 56 and compared to 12N group at days 14 and 28. Likewise, there was significantly higher number of calcified nodules (as measured by MRI) in (i) 12N group at day 28 compared to 12N group at day 14, (ii) 12N group at day 56 compared to 12N group at days 14 and 28 and (iii) 12N group at day 56 compared to 9N group at day 56. Histological analysis confirmed the mineralized nature of ectopic nodules as evident by von Kossa staining (Fig. 5E). Furthermore, the calcified nodules stained positively for both type I collagen and type II collagen. Type I collagen expression was predominately on the periphery of the nodules while type II collagen was expressed mainly in the center (Fig. 5F,G).

### PTOA development

**Figure 6:**
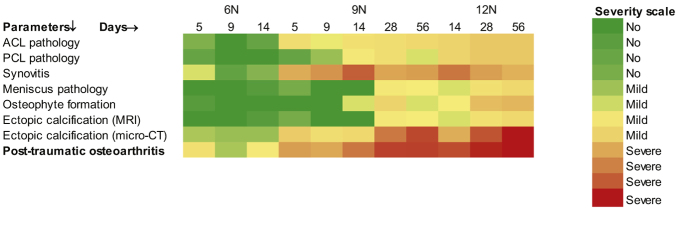
Summary. We generated heat maps of 12-tiered relative severity score of several parameters (e.g. ACL and pathology, cartilage degeneration, synovitis, meniscus pathology, osteophyte formation, ectopic calcification) to provide a summary knee PTOA. These heat maps indicated a load- and time-dependent increase in severity of PTOA. MRI = magnetic resonance imaging.

The individual parameters studied above clearly indicate total joint changes following loading. When we summed up the data from ACL, PCL and meniscus pathology, synovitis, osteophytes and ectopic calcification, we found that at 6N loading and early time points (up to day 14) there were subtle pathological manifestation which slightly increased with 9N loading and became severe at 12N loading and at late time points (Fig. 6).

## Discussion

Using a population genetics approach, we have previously reported that two recombinant inbred mouse lines, namely LGXSM-6 and LGXSM-33, differ significantly in their ability to heal ear wounds, to regenerate their knee articular cartilage and in their susceptibility to PTOA induced by destabilization of medial meniscus (DMM)^22^. In this study, LGXSM-6 and LGXSM-33 elicited no significant differences in healing or degeneration in this model of ACL injury: this may be attributed to the nature of model used here and the type of injury created. The DMM model^22^ is invasive and slow progressing in nature, whereas our ACL injury model is non-invasive in nature and results in knee joint changes within hours or days following injury. We also observed no significant difference in number of apoptosed cells between the two strains (data not shown), which is consistent with our recent observations in a separate study, where we found that LGXSM-6 had 13.50 ± 1.85 cells per 1.92 million square pixels and LGXSM-33 had 13.33 ± 1.19 cells per 1.92 million square pixels^20^. The lack of difference between the two mouse lines is largely consistent with our latest observations for cartilage phenotype and cell response after injurious loading in early time points. Although, we have observed some differences in synovitis at early time points in a separate study, we did not observe significant differences between the two mouse lines in the current study. Therefore, we have combined the data from these two mouse lines in an effort to gauge the response of the knee joint to injury and to provide a time course of PTOA, using different intensities of loading while tracking pathological changes across a series of time points.

We used a single session multi-cyclic loading pattern with the idea that this loading strategy will result in significant cartilage fibrillation and degeneration over an extended period, as we already know that cyclic mechanical stimulus results in focal cartilage damage within 14 days^10^. Christiansen and colleagues^7^ used a single-cycle loading strategy that induced ACL rupture and resulted in mild OA by 56 days, with the cartilage lesions localized in all of the compartments of the knee joint. In this model, the ACL rupture induced by the single-cycle load released energy that likely reduced the direct damage to the cartilage. Thus, a single bout of tibial compression (i.e. ACL rupture approach) induces joint destabilization rather than direct cartilage injury. Accordingly, cartilage degeneration seen in all compartments of the knee by 56 days was likely induced by habitual use following ACL rupture. In our loading strategy, there was more direct impact on the articular cartilage that resulted in direct cartilage injury. Thus, this loading pattern reflects human knee trauma to both cartilage and ACL, and PTOA. The purpose of the knee loading injury is to model the resulting trauma and not necessarily, the exact mode of trauma incurred on the knee. In addition, the mechanical loads employed here are more physiological than those by Christiansen and colleagues^7^, which supersede the levels ever experienced in the tibia^30^. Lastly, while it may seem that human PTOA is induced by a single traumatic event, it is typically the result of an accumulation of loading events as is used in our model.

We demonstrate that higher mechanical impact over a longer time scale results in distinct molecular changes characteristic of PTOA as have been defined elsewhere^31^. ACL tear, one of the primary outcomes of loading, instigated many, if not all, of the subsequent events (except for primary cartilage injury and chondrocyte apoptosis). We used MRI as a technique to assess soft tissue lesions with its superior soft tissue contrast^32^,^33^. ACL tear following loading as reported by us^10^ and others^7^ is generally appreciated by the abrupt release of compressive force, occasionally confirmed by histology. Here we have also measured the tear and pathology of ACL by MRI, which was highly consistent with the Instron recording. We also observed that PCL was injured by compression similar to ACL as measured by MRI. MRI assessment showed that both ACL and PCL in non-loaded knees were a continuous band of uniform intensity while in the loaded knees they appeared as thin and broken bands with varying intensities. From these observations, it is tempting to believe that PCL is effected in the same way (in terms of injury/transection) as ACL and follows the same pattern of pathological changes (i.e. increased pathology at early time points for 6N and 9N groups and at late time points in 9N and 12N groups). In contrast to previous studies on loading model^7^,^8^,^10^,^20^, which largely focused on ACL and did not report whether the PCL was effected or not, our study provides *bona fide* information on PCL injury.

MRI has helped us measure meniscus and PCL pathology, which would otherwise have been difficult to assess by histology alone. Although there were subtle differences in meniscus pathology across loading groups and time points, our findings of meniscus and PCL pathology in the mouse were consistent with the observations that they are associated with cartilage damage and OA in humans^34^,^35^,^36^. Additionally, our findings of gradual increase in osteophyte count over time (especially in 12N group) in loaded knees is consistent with another study of ACL transection in mice^37^.

A consistent finding is the identification of a secondary cartilage injury distant from the primary injury site. Although both injury sites are identical in characteristics, (loss of proteoglycan, apoptosis, altered aggrecan expression pattern), secondary injury exclusively occurred in association with an ACL tear, while the primary injury is thought to be a consequence of direct compression (anterior displacement of tibia and femoral-tibial direct contact). Unlike other studies that show cartilage fibrillation and loss of surface zone with chondrocyte apoptosis and onset of OA^7^,^38^,^39^,^40^,^41^, we failed to see appreciable cartilage fibrillation. In line with other studies, we observed loss of proteoglycan^7^,^10^ and presence and persistence of apoptosis^10^,^42^,^43^ at the site of cartilage lesions. However, lack of progression in cartilage degeneration was quite unexpected. Although, it has been reported that mechanical loading for extended time period induces cartilage fibrillation, fragmentation, and erosion^11^, Poulet *et al*.^9^, reported that a single loading episode does not induce progression in articular cartilage lesions. A recent study by Zhang and colleagues^44^ provides a plausible explanation for this disparity in observations. In this study, the authors induced autonomous expression of diphtheria toxin to kill articular surface chondrocytes in mice and concluded that chondrocyte death neither leads to cartilage damage nor drives its progression in response to injury. These observations suggest that chondrocyte catabolism, not death, contributes to articular cartilage damage following trauma.

While we observed the presence of apoptosis at early time point (day 5), at later time points, there was no further apoptosis, indicating that the cells died at the site of impact and no additional cells underwent apoptosis thereafter. This is coupled with the observation that the cell death occurred at loading and did not persist past day 5. Apoptosis and substantially low number of living chondrocytes in the calcified layer apparently do not impair cartilage under normal conditions^27^,^28^. Therefore, we only compared chondrocyte apoptosis in the impacted area of non-calcified cartilage. Other studies have shown an acute chondrocyte necrosis and apoptosis in response to blunt trauma to cartilage^43^. Our findings of apoptosis in the injured cartilage are in line with our previous observations^10^,^20^,^21^ and with other studies^45^. Furthermore, our unpublished data suggest that apoptosis begins as early as 12 h post-loading (Duan X, Rai MF, Sandell LJ). The mechanism of aggrecan internalization in the apoptotic cells has been described by us^10^,^20^. We have reported that, in the loaded knee, aggrecan was found in the injured chondrocytes, co-localizing with the TUNEL staining. We further investigated whether this intracellular aggrecan was intact or fragmented, or newly synthesized. To this end, immunostaining for the C-terminus of the aggrecan (COOH-) cleavage product, NITEGE, revealed a higher degradation of aggrecan matrix in the intact cartilage (adjacent to the injured area) in the loaded knees compared to control knees. In the injured area, NITEGE was mostly detected in extracellular matrix but not in the injured chondrocytes, where aggrecan and Safranin-O staining was lost. This finding indicated that injured chondrocytes did not take up the cleaved aggrecan molecules while intact chondrocytes are responding to the injury by taking up the fragmented aggrecan^20^,^46^. Lack of strain differences in apoptosis suggests that genetic differences do not necessarily link to greater susceptibility to mechanical damage in cartilage^47^ at least in these mouse strains.

Synovium is believed to participate in OA pathogenesis^10^,^48^,^49^ and its involvement in OA is often accompanied by infiltration of the sub-lining tissue with scattered foci of inflammatory cells and eventually osteophyte formation^50^,^51^,^52^. Although we have shown that synovitis and cartilage degeneration are uncoupled^23^, others have suggested their co-occurrence^53^. We have recently shown that ectopic calcification has a strong genetic component to it as evidenced by identification of quantitative trait loci in the advanced intercross population of LG/J and SM/J^54^. Using LG/J, SM/J and their advanced intercross, we previously discovered that joint destabilization instigates meniscal and synovial ectopic calcification^54^. Interestingly, we observed the same phenotype in loaded knees and more so in the knees with ACL tear (data not shown), thus strengthening our hypothesis, that calcification occurs in unstable joints. Our data showed increased severity of ectopic calcification with loading intensity (9N vs. 12N at day 56) and time (day 56 vs days 14 and 28 in 12N group) indicating its role in PTOA.

Among the tibial trabecular bone parameters and subchondral bone plate thickness, we observed a subtle change in trabecular BV/TV, Tb.N, Tb.Th, Tb.Sp, trabecular CDI (a measure the number of trabecular connections) and apparent BMD. We observed significantly higher SMI (an indicator of shape of trabeculae) with loading (at day 14) but not across other time points. Our finding of higher SMI (cylindrical rod-shaped trabeculae) as a marker of PTOA is in line with several other studies^55^,^56^. Overall, these differences reflect trabecular bone structure with thinner, more widely spaced trabecular struts following 9N and 12N loading intensities. There were no obvious changes in the subchondral bone plate thickness in 6N and 9N groups. Interestingly, however, there was significant subchondral bone plate thickening at day 56 in 12N group compared to early time points, which was consistent with our previous observations in DMM model^22^.

We used contralateral limbs as internal non-loaded controls, which is not considered ideal since this limb may be exposed to systemic effects of the injury and may also experience altered loading if the ipsilateral leg is used less by the mice. As it has been reported that non-loaded contralateral limbs show some alterations in subchondral bone plate thickness and epiphyseal trabecular bone mass^57^, we considered the use of contralateral limb as a potential limitation in this study. However, the reported study used multiple loading strategies, the changes were confined to various bone parameters and were transient. In our study, we did not observe significant alternations in the contralateral limbs which is consistent with other studies that support the use of contralateral limbs as non-loaded controls in mice^58^ and rats^59^. Another limitation in our study is the lack of data on all time points across all loading groups. Taken this limitation into account, the conclusions were drawn with great prudence. Our conclusions are not limited to temporal pattern of disease progression at different loading magnitudes but we also considered the differences between 6N and 9N groups at days 5, 9 and 14 and between 9N and 12N groups at days 14, 28 and 56. It means that the significant effects are being driven by comparisons of loading intensities at same time points rather than at the different time points and the assessment of disease progression over time is made using comparisons between different injury groups within same time points. Lastly, in this study, we did not measure OA biomarkers, which would indicate molecular-level changes in the joint following mechanical injury. At the same time, we believe that at this early stage with so little cartilage degradation or bone changes, it would be unlikely to see any changes in the traditional biomarkers in mice. In addition, OA biomarkers for mice have not been sufficiently validated. Despite these limitations, actual studies along these lines would shed further light on the utility and validation of biomarkers in mouse model of PTOA.

Taken together, this study provides a broad picture of pathological events in the mouse knee following a traumatic insult strong enough to sever the ACL. We observed that high mechanical loading instigated whole knee joint changes leading to PTOA. At least three phenotypes, namely chondrocyte apoptosis, synovitis and ectopic calcification, are targets for potential therapeutic intervention. Although the actual mechanisms underlying these changes in mice and men may be alike, the time scale for progression to advanced PTOA is greatly accelerated in mice as compared to humans; therefore, translating these findings to human PTOA should be undertaken with great sagacity. Further interrogation of the mechanism(s) of these changes is warranted in specific gene knockout mice to unravel the exact pathogenetic pathways of PTOA.

## Additional Information

**How to cite this article:** Rai, M. F. *et al*. Post-Traumatic Osteoarthritis in Mice Following Mechanical Injury to the Synovial Joint. *Sci. Rep.* **7**, 45223; doi: 10.1038/srep45223 (2017).

**Publisher's note:** Springer Nature remains neutral with regard to jurisdictional claims in published maps and institutional affiliations.
